# Intuitive real-time control strategy for high-density myoelectric hand prosthesis using deep and transfer learning

**DOI:** 10.1038/s41598-021-90688-4

**Published:** 2021-05-28

**Authors:** Simon Tam, Mounir Boukadoum, Alexandre Campeau-Lecours, Benoit Gosselin

**Affiliations:** 1 Department of Electrical and Computer Engineering, Université Laval, Québec, G1V 0A6 Canada; 2 Department of Computer Engineering, Université du Québec à Montréal (UQÀM), Montréal, H2L 2C4 Canada; 3 Department of Mechanical Engineering, Université Laval, Québec, G1V 0A6 Canada; 4 Center for Interdisciplinary Research in Rehabilitation and Social Integration, Québec, Canada

**Keywords:** Machine learning, Skeletal muscle, Quality of life, Data processing, Computational models

## Abstract

Myoelectric hand prostheses offer a way for upper-limb amputees to recover gesture and prehensile abilities to ease rehabilitation and daily life activities. However, studies with prosthesis users found that a lack of intuitiveness and ease-of-use in the human-machine control interface are among the main driving factors in the low user acceptance of these devices. This paper proposes a highly intuitive, responsive and reliable real-time myoelectric hand prosthesis control strategy with an emphasis on the demonstration and report of real-time evaluation metrics. The presented solution leverages surface high-density electromyography (HD-EMG) and a convolutional neural network (CNN) to adapt itself to each unique user and his/her specific voluntary muscle contraction patterns. Furthermore, a transfer learning approach is presented to drastically reduce the training time and allow for easy installation and calibration processes. The CNN-based gesture recognition system was evaluated in real-time with a group of 12 able-bodied users. A real-time test for 6 classes/grip modes resulted in mean and median positive predictive values (PPV) of 93.43% and 100%, respectively. Each gesture state is instantly accessible from any other state, with no mode switching required for increased responsiveness and natural seamless control. The system is able to output a correct prediction within less than 116 ms latency. 100% PPV has been attained in many trials and is realistically achievable consistently with user practice and/or employing a thresholded majority vote inference. Using transfer learning, these results are achievable after a sensor installation, data recording and network training/fine-tuning routine taking less than 10 min to complete, a reduction of 89.4% in the setup time of the traditional, non-transfer learning approach.

## Introduction

Myoelectric hand prostheses offer a way for upper-limb amputees to recover gesture and prehensile abilities to ease rehabilitation and daily life activities. Using surface electromyography (sEMG), non-intrusive wearable prosthetic devices are designed to be easily accessible. They often require custom physical fitting to the limb, but avoid permanent surgery. Popular bionic prosthesis manufacturers such as *Ottobock* (Germany), *Össur* (Iceland) and *Openbionics* (United Kingdom) offer highly attractive solutions in term of the mechanical hand itself and its motion functionalities. However, their lack of an intuitive control interface is often cited among the main limitations and user concerns towards prosthesis devices^1,2^. Their trigger-based control system generally uses one or two sEMG channels to map single muscle contraction events to pre-recorded movement sequences of the prosthesis^3–5^. This process requires an explicit mode switching command from the user to specify which grip mode to execute. Not only is this routine non-intuitive, but it also induces considerable latency in reaction and execution time as multiple separate commands are required to transition from one hand grip state to another. The upside is that safe operation is guaranteed, since the limitations ensure that false positives, i.e. wrong grip activation, are avoided.

To address intuitiveness issues, prototype solutions in the literature target specific finger flexor and extensor muscles in the forearm to decode amputated user gesture intentions^6,7^. When the amputation severity allows it, voluntary contraction of the resilient forearm muscles can be recognized by a classifier, thus providing the target command for the mechatronic hand to mimic the intended gesture^8,9^. However, targeting specific muscles requires precise electrode positioning, which is critical to feature-based approaches^7,10^. This may be hard to achieve consistently by end users in a real world scenario, resulting in degrading recognition accuracy and the necessity of training the algorithm over again.

The latest advances in myoelectric control have focused on advanced learning algorithms and spatially structured sEMG^11,12^. Notably, high-density electromyography (HD-EMG) allows to extract patterns in the spatial distribution of motor unit action potentials (MUAP) for different gesture-specific muscle contractions^13–16^. This method uses an array of electrodes placed over the forearm, reducing the installation process to a single sensor device instead of individual electrodes. Furthermore, using processing algorithms such as a convolutional neural network (CNN) alleviates sensor placement reliance, thanks to the CNN’s property of translational invariance^17^. This is in addition to the CNN’s ability of automatic feature extraction, which allows for an intuitive control interface, as intended gesture commands are decoded directly from the user’s natural muscle contraction patterns. This concept was proven in Tam et al.^18^, where the feasibility was demonstrated for a completely embedded deep learning workflow, including training and real-time inference pipeline. The wearable HD-EMG sensor was presented initially in Tam et al.^19^, with an improved version introduced in Tam et al.^20^. The latter contribution also presented an offline validation of the system with 8 users. The custom sensor fits in the socket format of common commercial myoelectric prostheses, ensuring straightforward device installation without added complexity related to HD-EMG sensing.

This paper investigates the deployment of a deep learning-powered neuromuscular interface for use in a personal biomedical device. This kind of application requires both reliability and practicality from a user’s perspective. Generally, a large amount of data is the key to unlock the capabilities and full potential of deep learning algorithms. As such, it appears incompatible with personal devices where the burden of data can be too demanding on the end user, let alone medically impaired patients. Addressing this concern, this work builds upon the system in Tam et al.^18^ by incorporating a 2-phases transfer learning (TL) approach in the CNN learning for substantially less network training time and training data from the end user (an 89.4% reduction of the prosthesis setup time). This allows for a new setup and fine-tuning method providing a more consistent and easier to train model through multiple sessions. Additionally, a new and thorough real-time study is conducted to properly evaluate the trained algorithm’s performance in response to the dynamically adaptive user input in the loop. In experimental tests conducted with a group of 12 able-bodied participants, time-distributed inference, motion selection time and positive predictive value (PPV) are used as metrics to assess and demonstrate the real-time control capabilities of the presented solution. Hence, this research’s contribution is a highly intuitive and responsive gesture recognition system for myoelectric hand prosthesis control. This paper demonstrates the successful deployment of a real-time deep learning pipeline in a personal wearable device, with a state-of-the-art setup that takes less than 10 min to complete. Therefore, this work proposes a realistic solution to end users seeking an easy-to-use intuitive control interface without sacrificing setup time/simplicity and reliability.

The paper is organized as follows: The Methods section presents an overview of the HD-EMG approach with the CNN and transfer learning implementation. A clear view of the real-time system loop involving the user is then presented to highlight the importance of appropriate evaluation metrics to better assess performance. The Results section then presents the experimental results for training/calibration time, real-time predictive accuracy and response time. Finally, the Discussion section reviews these results, the research limitations and future work.

## Methods

**Figure 1:**
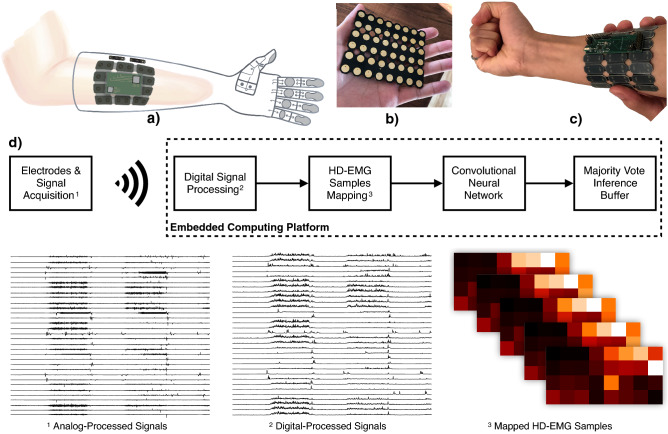
Prosthesis system concept and hardware components. (**a**) Integrated hand prosthesis concept. (**b, c**) Bottom and top view of the electrodes array and the data acquisition platform. (**d**) Block diagram of the myoelectric prosthesis control solution. The user’s voluntary muscle contractions are sensed by a 32-channel high-density electrode array. After analog filtering and analog-to-digital conversion, the signals are sent through a wireless link to the computing platform. The signals are then processed by a mean absolute value (MAV) filter and the samples from each channels are mapped into 4x8 matrices. The resulting muscle activation maps are fed into the convolutional neural network and the inference results are stored in a buffer from which a majority vote is extracted to provide the final gesture recognition output. Drawing in (**a**) was produced by *Agence IMPAKT Scientifik Inc.* following specifications from the researchers.

The presented solution involves a 32-channel electrode array, a signal acquisition interface, an embedded computing unit and a battery power supply. These hardware components and the envisioned prosthesis system are detailed in Tam et al.^18^ and Tam et al.^20^. Figure 1 displays the system’s concept and hardware components along with a block diagram where the evolution of the signals and data in the pipeline can be visualized.

### High-Density Electromyography and Wearable Sensor

Signals from the 32-electrode HD-EMG sensor are interpreted as a continuous flow from a 4 by 8 data frame as depicted in Fig. 1d, with each unit in the frame corresponding to the sample from a specific EMG channel. Individual channels are processed with band-pass and mean absolute value filters before mapping to the data frame as described in Tam et al.^20^.

Traditionally, time and frequency-domain features are extracted from individual EMG channels. With the HD-EMG approach, an additional dimension of features can be leveraged by using the spatial distribution of muscle activity captured by the array sensor. Furthermore, building the electrodes into a single apparatus simplifies the installation, compared to positioning multiple individual electrodes.

Typical myoelectric prostheses are worn by fitting the amputated limb in a socket^4^. Mechanisms such as tightening the device around the arm and/or custom shaped enclosures ensure a solid but comfortable wear. By installing dry electrodes on the inner walls of the socket, their positioning on the limb can thus stay consistent during daily use and over several sessions. To preserve comfort and remain non-invasive, the dry surface electrodes employed don’t require any skin preparation, shaving or application of conductive gel.

### CNN and transfer learning

A convolutional neural network approach was preferred to extract information from the array sensor data. The CNN is a powerful tool for an adaptive prosthesis solution. During the training process, it autonomously extracts relevant features from the HD-EMG data frames. As a result, when it comes to tuning the network to individual users, their limb and neuromuscular specificity is captured from the acquired data to allow for a customized solution. The convolution layers also brings translational invariance to the neural network’s performance^17^, for maintained performance and feature relevance even with sensor displacement. The convolutional neural network was custom-designed for the problem and its architecture is detailed in Tam et al.^18^. In the end, learned feature extraction, along with a one-size-fits-all sensor, provides a powerful universal adaptive system for different amputees and their specific needs.

Generally speaking, the more data the better it is for a machine learning algorithm to perform well once deployed in production. This is the major downside of this approach for a user-specific system, as the burden of data is on the end user. Various gesture examples with multiple repetitions each must be recorded to train the algorithm, resulting in a tedious and lengthy process. In this situation, another key advantage of the presented deep learning approach is the possibility to leverage transfer learning to alleviate the data burden on the end user^12^. The principle is that the neural network’s knowledge of a task or problem can be relevant for a different but related problem, e.g., the classification of muscle activity data from different sessions and/or users. In these situations, the neural network should be able to leverage prior training, to a certain extent, and not have to learn all over again. In this paper, the goals of the transfer learning techniques employed are to reduce the burden of data and facilitate inter-session usability for the end user. The algorithm first learns to extract muscle contraction features from HD-EMG data and to classify the user’s performed gestures. This knowledge is then used as a basis for subsequent use sessions where the network only has to be fine-tuned to offer optimal performance.

In the initial phase, rather than ask the user to provide training data from multiple sessions, data from a single session is used in combination with a group of participants’ data. The idea is to provide a feature-rich dataset at the initial session without extensive data recording from the end user. First, the neural network is pre-trained on a multi-subjects HD-EMG dataset, which does not include the test subject. This provides a general network initialization encompassing multiple subjects, each one with his/her own specificity regarding limb size, muscle mass, resilient neuromuscular capabilities, etc. Then, for the neural network to adapt itself to the end user, a one-time training of all the layers of the initialized network is done with the its own dataset. The network is then ready to be deployed for the current session, or can be stored for use in a future session.

In subsequent sessions, for the same individual, knowledge extracted and learned by the network in past sessions is expected to be relevant. Thus, parameters of the first layers of the CNN, which are mainly feature extraction layers, are kept as-is and only the last layer is trained. The network classifier is effectively updated, given the new context, with a reduced amount of training time and data compared to a complete training.

This transfer learning process allows for quick initial prosthesis setup and subsequent fine-tuning for the amputee. Routinely updating the weights and biases of the classification layer, the prosthesis can adapt to improving user capabilities as he/she’s getting used to the control scheme and motor memory develops.

### Real-time prosthesis control

Myoelectric prosthesis control systems are akin to feedback control systems, especially when involving continuous EMG control rather than short muscle contraction triggers. The user’s gesture intention provides the target input command, which then goes through voluntary neuromuscular activity, EMG sensing, signal processing, classification or regression (if applicable), and motorized hand actuation. The operator is involved in the control feedback loop as the gesture recognition output can be monitored in real-time. Consequently, the user can adjust muscle contractions to compensate for errors in the classification or regression output. The work in Côté-Allard et al.^12^ demonstrated that users with visual feedback from the classifier output are able to maintain higher gesture recognition accuracy over time when compared to users without feedback.

**Figure 2:**
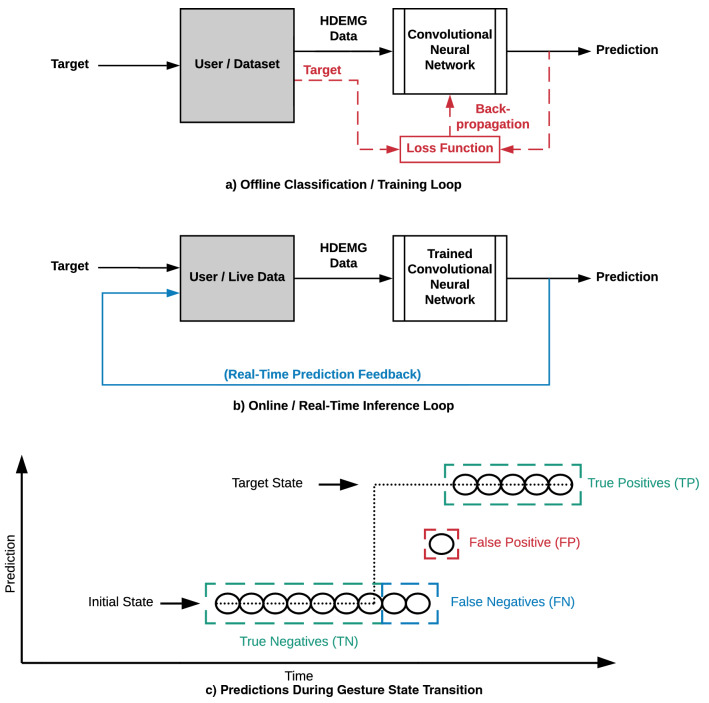
User interaction with the algorithm in offline and real-time scenarios. (**a**) Offline classification presents no user interaction with the algorithm. The user records a dataset, generating muscle contractions corresponding to target gestures. Data is then sent to the neural network with its output being compared to the recorded target label for training purposes. (**b**) The real-time inference loop allows for the user to react to the prediction feedback. Voluntary muscle contractions can be adjusted in order to maintain or change the predicted output. (**c**) Example of predictions during a gesture transition. Subsequent initial state predictions are considered true negatives (TN), and false negatives (FN) if the motion is engaged, but the prediction has not yet changed. Once a new gesture is predicted during the transition, the new prediction is considered a false positive (FP) if incorrect, or a true positive (TP) if correct.

Figure 2a,b illustrates the user’s involvement in the control loop. Two scenarios are depicted: offline and online. First of all, the neural network must be trained. This step is executed offline as the user records the data examples. The obtained dataset is then fed to the learning algorithm. This process is user blind, in the sense that no user feedback is given about the network’s output at the time of recording the sensor data; only the target gesture is given for the recorded muscle contractions performed. Hence, this training step doesn’t involve user intervention beyond the muscle contractions and is computed automatically from the dataset and the backpropagation algorithm.

Once the training phase is completed, the trained neural network is deployed for inference in production. In this scenario, the system takes the online form shown in Fig. 2b. As the data from the EMG sensor are fed into the neural pipeline, there are processes in real-time making a prediction from the muscle activity. The predicted gesture can be returned to the user as a visual label for instant verification. The user can then use the real-time prediction outputs to adjust voluntary muscle contractions to maintain or achieve the desired target prediction. In the integrated prosthesis system, the prediction will trigger control commands for the bionic hand motors/actuators to execute the gesture. In Hahne et al.^21^, the effect of the user in an online closed loop myoelectric man-machine interface was demonstrated. While the study was more conclusive with regression, instead of classification, the results show that users can adapt and regulate their muscle contractions successfully in reaction to error-inducing non-stationarities in the EMG signals. They also show that offline tests are insufficient to evaluate myoelectric control schemes. Thus, proper online performance metrics are necessary to evaluate the machine learning algorithm’s performance in conjunction with user adaptation^21–23^. In a classification system, rather than the continuous correction of contraction amplitude, the user can quickly identify true and false positives and adjust muscle activation patterns for future inferences accordingly. Over time, motor memory can develop to help provide better consistency and reaction time from the user voluntary contractions. In Hargrove et al.^24^, the subjects showed substantial improvements after practice using a myoelectric pattern recognition prosthesis in a 6 to 8 weeks home trial. This can be attributed to the notion of evolving user skill^25^ with the control system, which should be accounted for in performance evaluation metrics and protocols.

### Data recording protocol

In Tam et al.^20^, the presented neural network was validated offline on 8 participants. In this paper, 6 of the previous 8 participants were recruited again for the transfer learning and real-time trials. Additionally, 6 new participants were recruited and added to the study. The experiments were conducted with this group of 12 able-bodied participants, aged from 24 to 44 years old with no known medical condition. Written, informed consent was obtained from all participants. All experiments described in this study were performed in accordance with relevant guidelines, regulations, and experimental protocols approved by the Laval University Research Ethics Committee (Comité plurifacultaire d’éthique de la recherche de l’Université Laval, approbation number: 2019-268 phase 1 / 01-10-2019).

**Figure 3:**
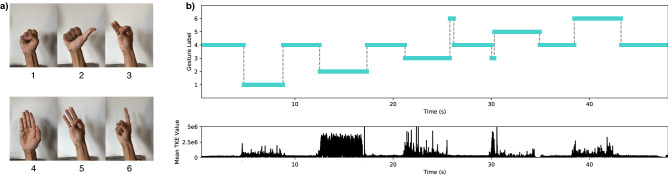
Real-Time Classification. (**a**) Set of 6 functional hand postures/grip modes: (1) closed hand/fist, (2) thumb-up and hook grip, (3) tripod pinch, (4) open hand/neutral position, (5) fine pinch, and (6) pointed index. (**b**) Time-distributed system outputs during a real-time inference test performed by user 3. The user cycles through every gesture, holding them for a few seconds and resting in the neutral position (label 4) in between. The top plot shows the system’s predictions (neural network + majority vote). The bottom plot displays the aligned mean Teager-Kaiser Energy (TKE) value to give an idea of the system’s responsiveness to the muscle contractions.

The set of 6 gestures retained for the current study is pictured on Fig. 3a. It is specifically curated from the typically useful functional hand prosthesis grip modes^4,26^. It reflects common prosthesis user needs and provides the functionality expected in a commercial prosthesis.

The initial protocol for building the training data set consisted in recording the user while holding every gesture for at least 5 seconds, in the order shown in Fig. 3a^20^. This cycle is repeated 5 times, which makes for at least 6 gestures × 5 seconds × 5 repetitions = 150 seconds of recording time. The same amount of time has to be accounted for off time in between gestures to minimize muscle stress and fatigue. A 2-min break was also imposed after 3 cycles for the user to relax before the last 2. With about 3 more min for the installation process of the sensor cuff, the entire process requires at least 10 min of active user involvement. The installation process itself consists of placing and fastening the sensor on the forearm. This can be done in less than one min, but to ensure good skin-electrode contact, and thus impedance, the sensor cuff is left sitting on the limb for two min before recording the data.

The results from the previous study^20^ showed, for 8 able-bodied participants, a median offline recognition accuracy of 99.57% and a median training time of 44.59 min on CPU. Thus, from donning the sensor cuff to testing real-time recognition, the whole process is expected to take 10 min of preparation and data recording plus the median 44.59 min of training (on CPU), for a total of about 55 min. The computer used for training had a 2.2 GHz Intel Core i7 CPU and 16 Go of 1600 MHz DDR3 RAM. The training times were evaluated on a CPU rather than a GPU to better reflect the current embedded prosthesis systems, as they typically don’t use a GPU for training purposes due to limited resources and power consumption constraints.

With this paper’s proposition to leverage transfer learning, the data recording protocol, for subsequent use sessions with a given user, is reduced to 6 gestures × 2 seconds × 3 repetitions = 36 seconds of recording time. Doubling that amount of time to account for off time in between gestures yields 72 seconds for the data recording process. With the device installation time of approximately 3 min, the entire process takes 4.2 min of active user involvement. For all experiments, the data sets were split the same way as the previous study, with 70% data for training, 15% for validation and 15% for test, with equal proportion of each target gesture.

### Evaluation metrics

A real-time gesture recognition algorithm’s performance during state transitions is critical to the applicability of a prosthesis control system in a clinical setting. This is especially true in pattern recognition and classification problems, where the algorithm is typically trained on steady-state hand posture contractions^27^. In these cases, gesture state transitions present ambiguous data that can be problematic to the classifier’s behavior. Appropriate evaluation metrics must thus properly account for these transitions and reflect the overall performance perceived by the end user.

Multiple real-time metrics are suggested in Xu et al.^28^. Time-distributed classification results are used to extract motion completion and selection times, motion completion rates, and real-time accuracy. In this paper, precision or positive predictive value (PPV) is preferred for its added nuances to accuracy. PPV evaluates the proportion of true positive (TP) predictions, given their number and the number of false positives (FP):

1 $$\begin{aligned} PPV = \frac{TP}{TP+FP} \end{aligned}$$

The metric is applied to the time-distributed algorithm outputs of a complete trial. This encompasses both the transient and steady-state contractions in order to provide a measure of the total real-time recognition accuracy expected over an “initial-to-target-to-initial state” gesture contraction sequence. The PPV metric allows to better handle the classification results during transients, i.e. gesture transitions. In the steady state, measuring time-distributed accuracy works well as the predicted values are evaluated against a single target. During the transition period between gestures, however, two different predictions can be valid depending on the time of occurrence. Thus, the goal is to detect if there is a gesture change, and if so, validate that the predicted value is accurate. Gesture transitions are evaluated on a binary basis:

- A **true negative** is a correct prediction when holding the initial gesture state.
- A **true positive** is a correct prediction on the new target gesture state.
- If the target changes, i.e. the user initiates a gesture transition, but the prediction is still the previously held state, it is considered a **false negative**.
- If there is a change of target detected in the transition, but the prediction is incorrect, it is considered a **false positive**. The logic is that while the transition is under way, the previous state should be held. The prediction should be updated once the transition is completed, i.e. once the voluntary contraction has reached its target.

False negatives are acceptable in a prosthesis control scenario since it can be perceived as latency. Furthermore, unresponsiveness from the prosthesis, i.e., holding the current gesture, is an arguably less critical error than the prosthesis executing a false movement. The proposed real-time PPV evaluation metric is thus designed to better reflect the perceived performance by the end user when a gesture transition is being predicted. Using PPV rather than plain accuracy allows to distinguish inaccuracy from latency induced by false negatives that occur in transient contraction events. Figure 2c illustrates what output is considered a true/false positive and true/false negative.

To quantify the overall responsiveness of the gesture recognition system in a non-ideal real-life scenario, a motion selection time (ST) metric is used in addition to the algorithm’s computational latency. Proposed in Xu et al.^28^, the ST is measured as the time from muscle activity onset to the first correct classification. In this work, the first correct classification is considered at the system-level output in order to represent the response time perceived by the user. Thus, the ST spans from the onset of movement towards the target gesture to the first correctly recognized label at the output of the majority vote process. A motion completion time (CT) is also suggested as the time from the beginning of the movement to the tenth correct classification. However, the presented system generally has a 100% recognition accuracy in the gestures’ steady state. CT is thus redundant with ST since it is a constant 9 sampling periods longer.

**Figure 4:**
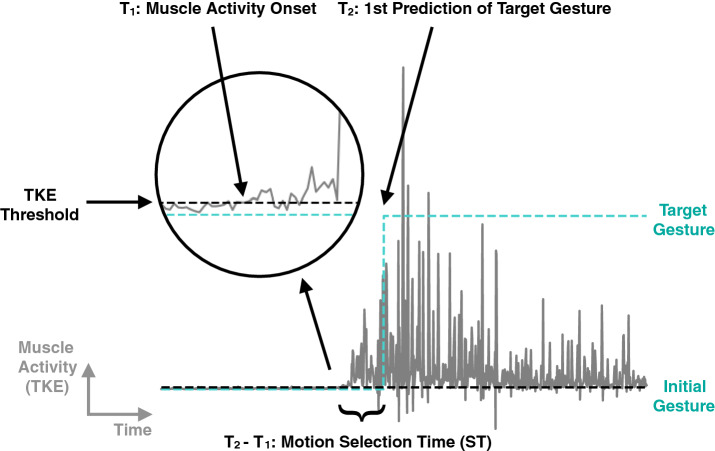
Motion onset detection with the Teager-Kaiser Energy (TKE) operator. The operator is used to monitor muscle activity and define a threshold to detect the onset of motion. The time from the onset to the new target prediction is referred to as the motion selection time (ST). This is the time it takes for the user to execute a target gesture and the algorithm to update its output accordingly.

In order to identify motion onset consistently, the Teager-Kaiser Energy (TKE) operator is used^29^. Illustrated on Fig. 4, a threshold is set to the mean + 2.5 × the standard deviation of the TKE noise floor before motion onset. After signal and noise analysis, this threshold has been set in order to detect voluntary contractions as soon as possible.

During the experiments, the positive predictive values and motion selection times were measured on 5 trials for each gesture class. The trials consisted of holding the gesture for 3 to 5 seconds, starting and ending on the rest position. This way, the muscle activity onset can be clearly monitored to identify transitions and measure response time. The neutral hand state (label 4) being a particular case, its real-time accuracy was measured during the resting phases in-between the contraction trials for the gesture label 1. No response time was measured for the neutral state since it produces no muscle activity.

## Results

### Training and calibration time

Leveraging transfer learning to facilitate inter-session algorithm training, the setup time for real-time operation is reduced by 89.4% on subsequent sessions. Concatenating the data from the 8 users of the previous study^20^ provided a one-time base training for the neural network, which took 252.68 min on CPU, or 4.21 hours.

For their first contact with the system, new users record training data with the initial, longer, protocol (5 repetitions of 5 seconds per gesture). Using the pre-trained network as an initialization for the end user, its dataset is used to complete the training of all layers. This step took a median training time of 13.30 min with the new users of this study. Adding the data recording time described in the Methods section, it makes for a total setup time of 10 + 13.30 = 23.30 min. This process only has to be done one time per new user.

Upon subsequent sessions, the training data recording protocol is reduced as described in the Methods section (3 repetitions of 2 seconds per gesture). Using this new dataset, training the last layer of the initially trained neural network took a median 1.58 min and achieved a 97.17% median test accuracy (with a majority vote over 5 successive inferences) for the group of 12 participants. The new total setup time is thus 4.2 + 1.58 = 5.78 min. This is an 89.4% reduction from the previous 54.59 min for individual training without transfer learning, as described in the Methods section. Offline accuracy has also been maintained with the new 97.17%, compared to the previous 99.57%.

Table 1 summarizes the median transfer learning and non-transfer learning setup times and accuracy observed with the study’s participants. With comparable classification accuracy in offline testing, the transfer learning setup routine takes less than 10 min, compared to almost an hour for the non-TL process. This brings the setup time down to a reasonable amount that can be completed as a daily routine by the end user without too much inconvenience.

**Table 1:** Transfer learning setup time improvements for subsequent sessions.

Median offline accuracy and setup times for TL and non-TL processes		
	Non-TL	TL
Sensor installation time (min)	3	3
Data recording time (min)	7	1.2
Network training time (min)	44.59	1.58
Total setup time (min)	54.59	5.78
Single vote accuracy (%)	92.28	84.78
Majority vote (n = 5) accuracy (%)	99.57	97.17

### Real-time classification accuracy

**Figure 5:**
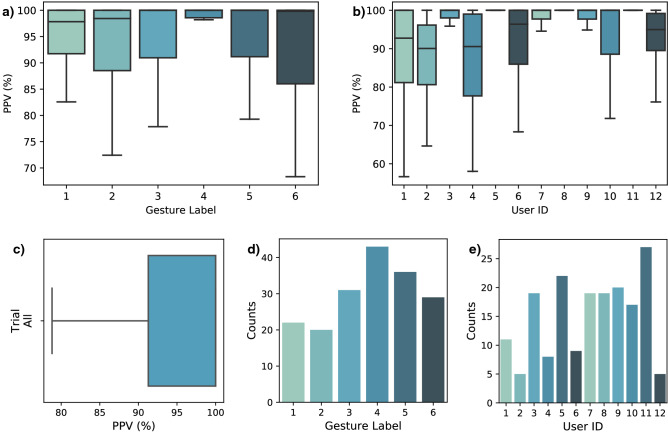
Real-time accuracy results. (**a**) Real-time trials evaluation results shown per gesture label and (**b**) per user. (**c**) Results of all trials across all participants shown on a box plot. The median positive predictive value (PPV) is 100%. The mean PPV is 93.43%. (**d**) Counts of perfect trials (100% PPV) per gesture and (**e**) per user.

**Figure 6:**
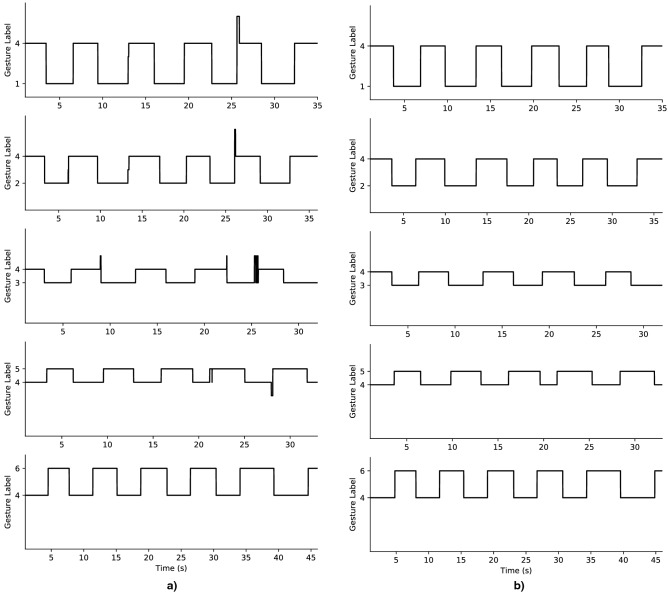
Real-time gesture trials of user 3. The user starts in a resting position (label ’4’) and completes 5 repetitions for each target gesture. (**a**) Trials with majority vote (200 inferences voting window). (**b**) Trials with thresholded majority vote (200 inferences voting window). A threshold of 102 votes has to be reached for the majority inference to update the output prediction. This results in 100% positive predictive value (PPV) for all trials.

Figure 3b displays the system’s inference outputs during a real-time test performed by user 3. The user was asked to cycle through every gesture, resting in neutral position (labelled 4) in between each active positions. Figure 5a,b present the measured real-time accuracy (as PPV) per gesture and per participant. Figure 5c gives an idea of the distribution of these results for all trials of all users on a box plot. One trial corresponds to the resulting PPV from a single contraction event (from the starting position to the target gesture and back). Figure 5d,e display the counts of 100% PPV trials achieved per gesture and per participant. The total number of trials per user is 30. Figure 6 provides an overview of all the real-time trials for a given participant. For users 5, 8, and 12, one of the gesture classes was excluded from the results. The criterion for detecting those outliers is based on qualitative observation of the algorithm’s behavior, where a clear and explicable exception occurred. While the algorithm was inaccurate for the given class, its behavior was consistent and predictable. This is further explained in the Discussion section.

### Response time

Assuming ideal conditions, i.e. perfect muscle contraction signals yielding 100% accurate predictions from the neural network, the system recognizes a gesture transition accurately with a 101 to 116 ms latency. Every sampling period (1 ms), a prediction is produced by the CNN and is stored in a first-in first-out (FIFO) buffer. 200 predictions are accumulated as votes in the buffer and the label holding the majority is given as the system output. The majority vote is computed every time the buffer is updated, thus producing an output every 1 ms. In the ideal scenario, the user starts by holding the initial gesture state, filling the buffer with its label. When the user engages a different gesture, the new label is being fed to the buffer every 1 ms. The system keeps outputting the previous gesture label until the new one reaches majority in the buffer, which would be 101 votes out of the 200. Although unreliably, the majority could be reached quicker if misclassifications occurred during the initial state. That would lower the number of votes for the previous label, which has to be surpassed by the new label to reach majority. Thus, 101 ms latency is expected as the maximum latency from the classifier. Including the signal acquisition, processing and inference pipeline, the complete system’s response time has an additional 15 ms to take into account the transient response of a 15-sample mean absolute value filter. Therefore, the maximum algorithm response time, under ideal conditions, is specified at 116 ms. An in-depth description of the signal processing and inference pipeline is given in Tam et al.^18^.

**Figure 7:**
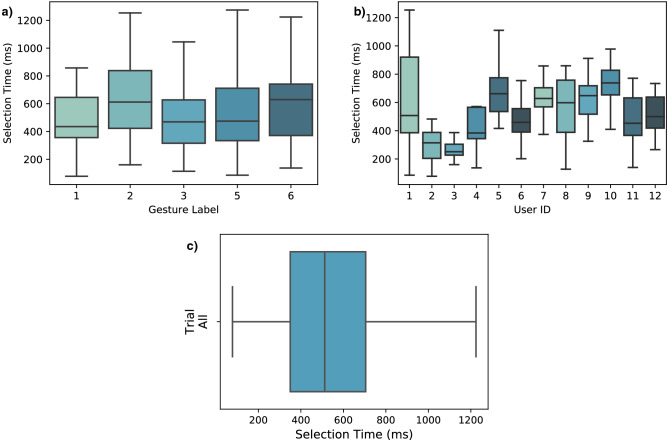
Motion selection time evaluation. (**a**) Motion selection time of the real-time trials per gesture label and (**b**) per user. (**c**) Results of all trials across all participants shown on a box plot. The median ST is 512.05 ms with a minimum reaching 78 ms. Selection times are shown for all gesture labels except label 4 since it is the neutral hand state used in all trials as a starting position.

As with the real-time accuracy results, Fig. 7a,b present the measured selection times per gesture and per participant. Figure 7c shows the selection times for all trials of all users on a box plot for an overview of their distribution. These results exclude the neutral hand position (label 4) since it produces no muscle activity onset allowing to measure the ST. The same outliers identified in the real-time accuracy results were excluded from the response time trials and are discussed in the following section.

Table 2 summarizes the theoretical and experimental response times of the presented system. Objectively, the algorithm has a response time of 116 ms under ideal conditions. Results will vary experimentally depending on the quality and speed of the voluntary contraction, but they should tend towards 116 ms as the user is increasingly skilled and experienced with the system.

**Table 2:** Summary of the system response time evaluation results. The algorithm’s theoretical response time under ideal conditions is 116 ms. Experimentally, sensor noise and the user’s voluntary contraction time contribute to the motion selection time, which has been measured at median of 515.9 ms with this study’s participants.

Response Time Analysis	
**Max. algorithm response time (ms)**	**116**
Fastest experimental motion selection time (ms)	78
Median experimental motion selection time (ms)	512.05

**Figure 8:**
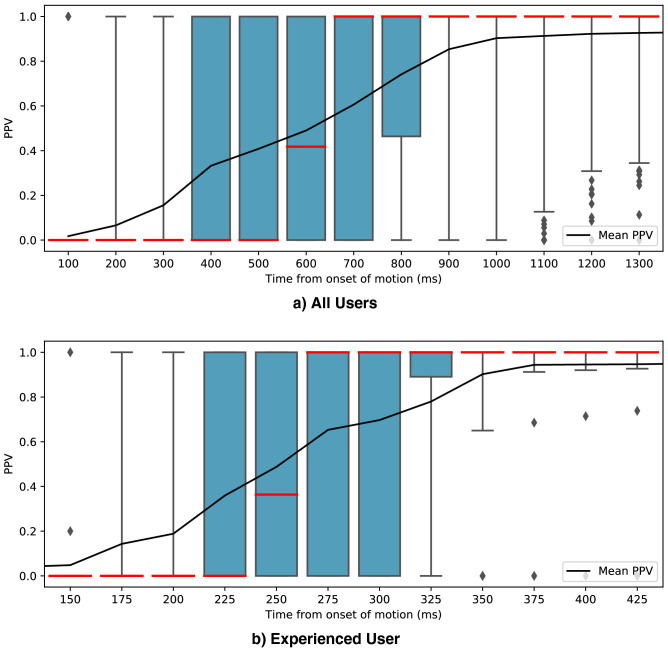
Positive predictive value (PPV) through gesture transitions. (**a**) Box plot representation of the PPV results from all trials of all users measured for different periods of time elapsed since onset of motion. (**b**) Box plot representation of the PPV results from all trials of an experienced user (user 3) measured for different periods of time elapsed since onset of motion. The whiskers of the box plots have been set to the 5th and 95th percentiles. The mean PPV is overlaid on top of the box plots.

In order to provide a comprehensive view of both the PPV and ST metrics, Fig. 8 presents the PPV measured for different periods of time elapsed since the onset of motion. Blue areas of the box plots represent the interquartile range with the whiskers displaying the 5th and 95th percentiles. Since PPV is undefined when there are no true and false positives measured, a default value of 0 is given in those cases to reflect that no accurate prediction occurred towards the target gesture. This happens when only true or false negatives occur, i.e., the algorithm shows no change in predicted output from the initial gesture through the transition.

## Discussion

Generally, as shown on Figs. 3b, 6, and 8, if misclassifications occur, they do during gesture transitions. In the former figure, the user cycles through all gestures and incorrect classifications were only observed during 2 gesture transitions. The first during the release of gesture 3, and the second during the onset of gesture 5. This trial was executed with little to no user practice, within a few minutes of the neural network’s training with transfer learning. A real-time display of the inferred label was provided as feedback to the user. As mentioned in the Methods section, the results are expected to improve as the user gets to practice regularly with the system and gets used to performing voluntary contractions more consistently.

With a mean and median PPV of 93.43% and 100% across all participants, the system proves to be highly accurate and reliable in real-time. Among the 12 participants, 3 have shown outlying results for a single gesture out of the set of 6. The outliers have been excluded from the reported results for both the PPV and ST evaluation metrics. For users 5 and 8, gesture class 2 was consistently recognized as class 1. For user 12, gesture class 5 was consistently recognized as class 3. As seen on Fig. 3a, those gesture pairs are relatively similar. Since those users were not physically impaired, the outliers were attributed to their inexperience with a myoelectric control interface^23–25^. This brings a probable inconsistency in the voluntary contractions during both the training and testing phase. Moreover, as mentioned in the Methods section, the synergistic relation between the user and the system in a real-time scenario depends on the user’s ability to continually adapt its contractions to the feedback from the algorithm. Thus, some users might need a prolonged period of practice to get used to the myoelectric control interface.

Looking further into the results confirms the potential of the system as every participant was able to achieve 100% PPV on a few trials as shown on Fig. 5d,e. Every gesture label has also seen 100% PPV trials. It should be noted that none but one of the participants were familiar with the gesture recognition interface beforehand. Thus, the current results can possibly be interpreted as a lower bound of the system’s performance, with higher real-time accuracy attainable with user practice. In fact, the highest overall results were achieved by the one user who had practice with the interface for several hours (user 3 with a mean 98.66 ± 1.29% PPV across all trials). Figure 6a shows an overview of the trials by this user. It can be observed that the classification errors in that experiment only occurred during some gesture transitions. This confirms that the user is able to produce flawless gesture executions and that errors in transition should reduce over time with practice.

While a 100% PPV can be expected reliably with user practice, it can also be achieved right away by the system, at the cost of response time. Currently, the majority vote inference is implemented in its most basic form: the label with the highest count is picked as the definitive inference output. By imposing a minimum threshold for the vote count to output a new label, the system refrains from making a new decision if the majority is weak. Implementation-wise, if the minimum vote count threshold is not attained, the system outputs the previous valid majority vote inference. This can be interpreted as the algorithm not being confident enough on a new decision, and thus maintaining the currently held gesture. As displayed on Fig. 6b, a threshold of at least 102, for a 200-votes majority window, yields 100% PPV across all trials of the same experiment displayed in Fig. 6a. With this mechanism, this experiment showed a mean motion selection time of 547 ±± 32.32 ms across all trials, compared to 269.77 ± 33.48 ms without.

Among all participants, the experimental ST is measured at a median of 512.05 ms, with the fastest trial at 78 ms. This time frame encompasses both the algorithm’s latency and the user’s voluntary contraction speed. Thus, results vary from user to user depending on their neuromuscular specificity and contraction speed, since no speed or strength was prescribed. This makes for a relaxed experience more akin to a real-life scenario. The best results were achieved by user 3 who had several hours of practice and is consequently more skilled with the system. The average selection time for this user was 269.77 ms. Slower results farther from the median are generally due to misclassifications before the target gesture is correctly identified. This is due to the motion selection time being defined by the time between the onset of muscle contraction and the first correct classification.

As with the real-time accuracy, these results can be interpreted as a lower bound of the system’s performance as most users had no prior experience with the control interface. Voluntary contractions can be performed faster as the user gets more comfortable and skilled with the system. This way, the ST can be reduced and tend towards the algorithm’s latency itself. To give an order of magnitude, Freund et al.^30^ investigated the mean contraction time for the voluntary contractions of human arm muscles on 6 subjects. The research reported a mean 88.3 ± 11.7 ms voluntary contraction time for an experiment consisting of performing isometric contractions and isotonic movements as fast as possible, with no target strength specified. This is similar to the current paper’s method, except that no speed was specified to the user. In this scenario, an estimation of the fastest motion selection time would add up the 116 ms max. algorithm response time to the 88.3 ms *fastest* voluntary contraction time, yielding 204.3 ms. This is less than half of the median 512.02 ms measured experimentally in the current study where the users performed slower voluntary contractions. In comparison, the more experienced user 3 achieved an average ST of 269.77 ms. To be taken with a grain of salt, this figure only serves as an estimation of the presented system’s potential if the users were to perform their muscle contractions reliably and as fast as possible. A reliable method to measure voluntary contraction speed would have to be employed in future experiments in order to properly isolate the user’s performance speed from the algorithm’s computational latency. Regarding the algorithm itself, its response time is in line with the 300 ms maximum latency prescribed in the literature^31–33^. The 116 ms maximum algorithm response time is also within the 100-125 ms optimal controller delay for myoelectric prostheses studied in Farrell et al.^31^.

In Fig. 8, the contraction trials are segmented with different lengths for the time windows, starting at the onset of motion (x-axis). Aggregating all trials of all users, this graph gives an overview of the gesture recognition performance during the gesture transition. As mentioned in the Results section, it should be noted that undefined PPV results were a possible occurrence due to the windowing. For instance, if a trial doesn’t yield any positive prediction during the initial 100 ms from onset of motion, the PPV result is undefined since no true nor false positives are observed. This is generally the case in the beginning of the motion as the system will output true and false negatives due to system latency and the quality and speed of the muscle contraction. In these undefined cases, a default value of 0% PPV is assigned to convey the information that no positive prediction is observed. Thus, results from Fig. 8 do not directly correlate with the previously reported PPV and ST metrics since the added default values affect the distribution. This also explains part of the variability in the results, as seen from the quartiles extending from 0 to 100%. This is due to the nature of the windowed results, which contain a dominant presence of 0% and 100% values. As seen in Figs. 3b, 5, and 6a, trials generally yield a 100% PPV during the gesture transition. Thus, when observing a segmented the trial, the earlier time windows will yield the default 0% PPV until the transition to the target gesture occurs. If no false positive is predicted, the PPV will be 100%. Overall, Fig. 8 provides insights on the potential performance that can be experienced with the system in real-time. For the experienced user on Fig. 8b, for instance, flawless detections can occur within 175 ms. Within 325 ms, 75% of the trials achieved a gesture transition at 90% PPV or more, with at least half being flawless.

In regards to the state-of-the-art, a fair comparison of gesture recognition controllers is hardly possible, with too many differing variables such as metrics, gestures, subjects and their specificity. This is especially true with real-time systems, where offline accuracy is often the reported metric even though it doesn’t properly reflect the system’s online performance. Roche et al.^34^ also made this observation while studying the barriers for the transition of multi-functional control algorithms from laboratory to clinical tests. The researchers concluded that speed and reliability of control are the key challenges to overcome for clinical use. The deep learning powered solution presented in this paper reaches the 100 % real-time accuracy (PPV) ceiling and the optimal myoelectric controller delay, all that with a setup time around 5.78 min. Thus, the next step is to pursue clinical trials of the system to properly evaluate its performance with the target user group^25^. While the potential of the system has been demonstrated in this paper, the variance and skewing of the results across the group of participant needs to be addressed in future work. This is due to the flexibility in the experiment protocol where each participant’s specificity was respected. They were free to perform the gestures in their own natural way without any precise guidance in terms of strength or speed. Since the amputees themselves show a wide-ranging specificity, an inherent variance in the results is to be expected. Flexibility might need to be retained in the protocol to ensure a proper case-by-case evaluation of the system and user performance. Experts in rehabilitation and medical sciences should be consulted to develop an experimental protocol as reliable as possible, while remaining flexible and suitable for amputees. New research will also be conducted to develop and implement real-time inference robustness mechanisms aiming to improve performance and reliability across all users.

At this point, the system has been proven on 14 able-bodied subjects offline and 12 in real-time with highly conclusive results for 6 hand gesture classes. After less than 10 min system setup, the real-time accuracy was measured during 5 repetitions of each gesture (3 to 5 seconds hold time) for all participants. With the system’s potential being demonstrated on able-bodied individuals, a major research limitation is the absence of assessment with amputees. This is a priority for future work. Qualitative appreciation also has to be surveyed among the participants, since the end goal is to improve the user experience and acceptance of myoelectric prostheses.

## Conclusion

Artificial intelligence (AI), especially deep learning, has been booming in the last years and has shown promises in a wide variety of applications and industries. In problems such as image recognition, data sufficiency is barely an issue with large aggregated data sets like ImageNet^35^ being available. However, when it comes to AI solutions personalized to end users, the burden of data can be a serious roadblock to real-life application. This is especially true in wearable systems intended to assist people with disabilities. The end user can’t be expected to provide a large enough set of example data for the neural network to learn from. That might be too demanding on the individual and, thus, hinder the device’s appeal. The presented solution to leverage transfer learning bridges the gap between the lab and real-life use of a machine learning powered myoelectric prosthesis. Reducing the amount of data and training time to less than 10 min (measured at 5.78 min in experiments, down from 54.59 min without transfer learning), the presented system makes no compromise on reliability and only requires a reasonable setup routine, time- and effort-wise, for the end user. The routine is short enough to be performed daily or spontaneously as needed without taking too much time out of the user’s day.

As observed in the inference time plots and the precision/positive predictive results, a high level of accuracy is achieved by the system in real-time. The results and inference plots show that 100% PPV is achievable by users with and without prior experience with the system. As the user gets to practice with the control system, better consistency in reproducing these flawless trials can be expected. This work’s results have also shown that implementing a threshold on the number of votes required for the majority voting inference can help achieve better robustness, particularly in gesture transitions.

The main contribution of this paper is the successful deployment of a real-time deep learning pipeline for hand prosthesis control that combines intuitive control, fast response time, user-friendly wearability and quick setup time. All these criteria aimed to address end user concerns in order to achieve higher user acceptance and satisfaction of myoelectric prostheses.

For this purpose, this paper proposes an intuitive myoelectric hand prosthesis control system that takes advantage of high-density muscle activity sensing and a real-time deep learning pipeline for high online accuracy. In particular, the paper puts a heavy emphasis on real-time performance demonstration with appropriate and transparent evaluation metrics to better reflect the device’s potential in a real-life scenario. The system is trained using a transfer learning approach to alleviate the burden of data on the end user. This has resulted in a setup time of less than 10 min to achieve a highly reliable real-time operation. 100% accuracy in real-time, evaluated through PPV, is achievable with a maximum algorithm response time of 116 ms under ideal conditions. These results demonstrate that the system is effectively bridging the gap between AI potential on paper and applicable real-life AI-powered solutions. With high performance and user-friendliness attained, clinical trials are next in line to further substantiate this solution’s claim for major improvements in myoelectric control.
